# Examining the Interplay between Queerness and Teacher Wellbeing: A Qualitative Study Based on Foreign Language Teacher Trainers

**DOI:** 10.3390/ijerph182212208

**Published:** 2021-11-20

**Authors:** Juan Ramón Guijarro-Ojeda, Raúl Ruiz-Cecilia, Manuel Jesús Cardoso-Pulido, Leopoldo Medina-Sánchez

**Affiliations:** Department of Language and Literature Teaching, Faculty of Education, University of Granada, 18071 Granada, Spain; jrgo@ugr.es (J.R.G.-O.); manueljcp@ugr.es (M.J.C.-P.); leoms@ugr.es (L.M.-S.)

**Keywords:** teacher wellbeing, LGTBIQ+, foreign language, teacher trainers

## Abstract

Oftentimes, teachers who identify themselves as LGTBIQ+ may feel unsafe at work, which may upset their wellbeing and destabilize their key psychological traits. Hence, feelings such as insecurity, lack of self-confidence, anxiety, and fear are on loop in their everyday lives. Thus, in this study we pursued an examination of the interplay between sexual orientation and teacher wellbeing in a cohort of seven university foreign language teacher trainers in a Spanish context. To gain insight into this issue, a qualitative study in line with the ecological paradigm was designed for the elaboration of semi-structured in-depth interviews and for the analysis of results. The main findings display teacher wellbeing as a complex interwoven system in which sexual orientation had played a core role in their identities, competences, private and professional relationships, and in the cultural and political spheres. We conclude by stating that although homophobic discrimination was a hard trial to overcome, the psychosocial capital of the participants allowed them to transform this negativity into positive assets such as queer activism in their private and political lives and in their profession as foreign language teacher trainers.

## 1. Introduction

Teacher wellbeing has received increased attention across a number of disciplines in recent years. With this being a trend in the field of applied linguistics [1,2,3,4], it is surprising that the wellbeing of foreign language university teachers has hitherto attracted scant attention by scholars worldwide. Except for Lander [5] and Lin, Trakulkasemsuk, and Zilli [6], studies are virtually non-existent. The population sample also has the particularity of being teacher trainers, which adds an engrossing perspective for the educational arena. Likewise, this research exposes the views of queer male teachers inasmuch as only these participants willingly decided to take part in our study. Therefore, this paper attempts to unveil how wellbeing is handled by seven queer foreign language university teachers in the context of Andalusia (southern Spain). To shed light on this issue, we carried out a qualitative study through in-depth semi-structured interviews with the aforementioned target group. It is worth noting that this research promotes three of the seventeen United Nations 2030 Sustainable Development Goals—namely, goal 3 “Good health and wellbeing”; goal 4 “Quality education”; and goal 5 “Gender equality”.

### 1.1. Study Background

Numerous studies in the international arena have brought to light the psychological and wellbeing challenges that LGTBIQ+ people must face in their professional and private lives to deal with the discrimination they suffer for questioning identity models imposed by the cisheteronormative system [7,8,9,10]. This situation seems to be worsened for those who are dedicated to teaching, already classified as one of the professions with the highest levels of stress and rate of attrition. For example, in the United Kingdom, up to 57% of teachers have considered abandoning the profession due to health problems derived from stress, work overload, or the impossibility of finding a healthy balance between work and private life [4]. Furthermore, we should add all the barriers that LGTBIQ+ teachers experience due to their sexual identity. Studies conducted in different global contexts point to the insecurity, anxiety, or fear that continuously erodes teacher wellbeing. This results in emotional and physical health problems and in a deterioration of teachers’ performance and professional competence [11,12,13,14].

In order to achieve quality education, we must pay attention not only to students, but also to teachers, who are an essential mainstay in the education system [15]. This situation remains contextually undertheorized in the field of foreign languages, where the advent of the learner-centered approach in the 1970s and 1980s placed the focus on what teachers could do for students and their teaching methods, competencies, or roles in the classroom. This sidestepped teachers as complex human beings, their identities, needs, interests, and their flourishing or floundering processes [1]. In this sense, this research casts light on how these teachers handle their teacher wellbeing ecosystem, so that they can flourish as individuals. This will also allow us to step out of the current educational dynamics and processes that robotize and dehumanize teachers [2].

If we compare the number of wellbeing studies focused on students with those of LGTBIQ+ teachers, the latter is still in the single digits [16,17]. These normally revolve around Primary or Secondary teachers, but little knowledge do we have about queer university teachers, with even less known if their area of expertise is foreign languages. This timely research fills a gap in the literature by interrelating the sexual identity of LGTBIQ+ teachers and teacher wellbeing from an ecological perspective. Key to this approach is the complexity of factors that affect teacher wellbeing in an interwoven architecture of five subsystems [4,18,19] that embrace multiple elements—namely, psychosocial capital, identity or professional skills, dynamism of perceptions and experiences about it over time, the culture of educational institutions, the interrelationships with the different agents of the system, and the cultural parameters of each geographic context. All of them play a pivotal role in teacher wellbeing, where teachers are central and proactive agents. Were they to know this structure and become aware of their own psycho-sociological capital, they would be able to develop strategies for the successful management of their wellbeing and power. Consequently, they would flourish satisfactorily in their work and life, teaching to the maximum of their potential, and being able to balance their physical and emotional health [18,19,20].

#### 1.1.1. The Concept of Teacher Wellbeing

The literature review carried out by McCallum et al. [21] highlights that there is no single agreed-upon definition of teacher wellbeing. However, and although wellbeing is multidimensional, according to the characteristics of our study, we understand teacher wellbeing as “a positive emotional state, which is the result of harmony between the sum of specific environmental factors on the one hand and the personal needs and expectations of teachers on the other” [22] (p. 286). By the same token, we agree with McCallum et al. [21] when conceptualizing teacher wellbeing, as their viewpoint respects the individual and foregrounds other features such proactiveness, humanity, and fluidity.

Wellbeing is diverse and fluid, showing consideration for individual, family, and community beliefs and for values, experiences, culture, opportunities, and contexts across time and change. Wellbeing is something we all aim for, underpinned by positive notions, yet it is unique to each of us and provides us with a sense of who we are, which is something that needs to be respected [21].

Next, we build upon existing research by suggesting those contributions that add significant nuances to the nature of our study. For some authors such as Deci and Ryan [23], wellbeing is twofold: hedonic and eudemonic. The former, widely known as subjective wellbeing, is characterized by defining it as the pursuit of pleasure and the avoidance of pain. The latter, eudemonic, focuses on meaning and self-realization, leaving the individual to use their own resources to grow personally [3,23]. Deci and Ryan [23] affirm that wellbeing fully resides within the individual, without being linked to external factors or contexts. Likewise, Oishi, Diener, and Lucas [24] indicate that subjective wellbeing is anchored and evaluated by each individual.

Day and Qing [25] explicate that wellbeing is a social and psychological construct—a dynamic state in which skills such as creativity and the creation of positive relationships with others stand out. In this vein, La Placa et al. [26] and Mercer [3] reveal that wellbeing is not only subjective but also objective and social. Gillet-Swan and Sargeant [27] add that wellbeing is a process, a capacity that is trained over time in order to manage situations, both constructive and unfavorable, and affects a person’s emotional, physical, and cognitive state. In the same way, Uchida, Ogihara, and Fukushima [28] assure that the understanding of wellbeing is context-bound to boot. These authors [28] distinguish between East Asian wellbeing (more focused on social harmony and compliance with social norms) and Euro-American wellbeing (concentrated on individual achievement and self-esteem).

Aelterman et al. [22] were pioneers in shifting the focus of teacher wellbeing. Previously, research often publicized the negative effects of low wellbeing, with stress, burnout, depression, and anxiety, often linked to attrition. These authors [22] demonstrated how the teachers’ experience is a cornerstone in the perception of their wellbeing (the greater the job experience, the better the perceived wellbeing). Likewise, Aelterman et al. [22] confirm that social support (or support in the workplace) is one of the most important factors when dealing with situations that alter professional and/or personal wellbeing. They [22] also placed emphasis on other elements that influence the teachers’ sense of wellbeing that are not part of the professional context, such as personality and personal background. These are likely to influence the way teachers cope with social demands and their own teaching praxis. Furthermore, they [22] carried out their study following a holistic teacher wellbeing model comprising three categories: personal, professional, and social aspects.

In this sense, Cook et al. [29] confirm that having high levels of teacher wellbeing reduce the perception of stress and improve teachers’ self-efficacy. Furthermore, Yin, Huang, and Wang [30] explain that trusting co-workers promotes wellbeing. Roffey [31] also pinpoints that resilience is a key factor in teachers’ wellbeing.

We espouse the reckoning of Jin et al. [4] when they state that “to teach well, teachers need to be well and to be well, they need to be individually and systematically supported to enable them to flourish and teach to the best of their abilities” (p. 31). Bearing this trend in mind, Diener et al. [32] include eight core dimensions of wellbeing: purpose and meaning, mutually supportive relationships, commitment and interest, contributing to others, competence, being a good person, optimism, and feeling respected. Therefore, it is worth examining teacher wellbeing from multiple holistic perspectives, paying special attention to social relationships [3] and to the interactions of teachers’ private and professional lives, since boundaries are usually blurred [33]. In the same way, Jin et al. [4] highlight the importance of investigating teacher wellbeing of novice, mid-career, and late-career stage teachers since the perception of wellbeing stems from each and every moment of a person’s life.

Over the last three decades, teachers’ burnout has attracted the interest of numerous experts in the fields of psychology and education as being a profession that often involves high levels of stress and low levels of professional wellbeing [3,20,34]. Grounded in recent research [3,4], it is agreed that teachers with high levels of wellbeing teach more effectively and that they are more creative, contributing positively to both their personal and professional growth as well as to the growth of their students’. Similarly, Gregersen et al. [2] reiterate that teachers’ educational wellbeing influences their abilities to build positive relationships with the various agents involved, reduce discipline and motivation problems, maintain physical and mental health, and teach to the maximum of their potential.

Our study adheres to a holistic approach in which we apply the ecological model of Bronfenbrenner [18] to our context. As we have emphasized, teacher wellbeing operates and establishes connections with numerous systems, which is why complex interactions between them are identified. Price and McCallum [19] and McCallum et al. [21] have also used this model to explore teachers’ wellbeing according to the systems proposed by Bronfenbrenner [18], finding a complex interplay between teachers’ wellbeing and their personal and professional lives [21]. In this line of research, the work by Jin et al. [4] stands out. They [4] analyze the wellbeing of compulsory education teachers using an ecological perspective. The systems they establish are:

- Microsystem: the classroom or the faculty where they teach.
- Mesosystem: work networks, interrelationships between different contexts (e.g., family and school).
- Exosystem: school organization and contextual influences.
- Macrosystem: broader spheres such as social, cultural, and political.
- Chronosystem: the significance of changes over time (e.g., in the teaching career, or the difference between being a novice or senior teacher).

Furthermore, these authors [4,21] show that teacher wellbeing, being in constant change and adaptation, must follow an ecological perspective. For this reason, they pay special attention to psychological and social capital. The former is described as a construct of positive psychology aimed at managing the psychological resources necessary to face the continuous challenges of the environment [4,20]. By contrast, the latter interprets teacher wellbeing as a social phenomenon. The greater the understanding, support, and recognition by society, the higher the levels of wellbeing [4]. However, despite that, the limits and resources of the ecological model are defined by the individuals themselves [34]; that is, the social capital varies according to each teacher since each perception is unique [4].

#### 1.1.2. Life and Work Conditions of LGTBIQ+ People

The declassification of homosexuality as a mental illness or disorder is a relatively recent event in human history. Let us remember that the American Psychiatric Association (APA) did not do it until 1973 and the World Health Organization (WHO) until 1992. In fact, the Inter-American Commission on Human Rights [35] continues to assert that discrimination of LGTBIQ+ people based upon their sexual identity remains one of the main causes of the violation of human rights in today’s world.

In this global context, Spain is considered one of the most legislatively advanced countries for LGTBIQ+ people in the world, allowing same-sex marriage since 2005 and enacting other educational bills that expressly ban discrimination based on sexual orientation. For example, in the region of Andalusia (southern Spain), where this study was carried out, the law to guarantee the rights, equal treatment, and non-discrimination of LGTBIQ+ people and their families was enacted in 2018 [36]. In addition, Spain is the European country that shows the most social acceptance towards this group according to the Pew Research Center [37], which points out that 88% of the population strives for homosexual rights. Thus, it is ahead of Germany (87%) or Canada and the Czech Republic (80%).

However, the Spanish Federation of Lesbians, Gays, Transsexuals, and Bisexuals (FELGTB) in its most recent report on hate crimes [38] portrays that associations received up to 971 communications of this type of crime in 2019. Of the victims, 70% are cis-gay men, and the age of up to 68% of them spans from 18 to 35 years old. The attacks are vastly underreported, inasmuch as they usually take place in the private sphere of the victims. These are often perpetrated by acquaintances or relatives, thus conspicuously increasing the vulnerability of this group.

In Spain, 7% of the total discrimination based on sexual orientation occurs within educational contexts. Furthermore, LGTBIQ+ phobia is, at the same time, the most common type of school bullying [38]. This is further aggravated since the school environment is supposed to be protected by public powers. In addition, we have to exercise a great deal of caution, as this is where the new generations are to be educated in values of respect and tolerance towards diversity.

According to the FELGTB [38], the root cause may lie in the social construction of a toxic masculinity that despises differences, the feminine, and all identities that diverge from the patterns of cisheteronormativity.

In another study conducted by FELGT-COGAM [39] with 703 LGTBIQ+ people, 45% responded as having been discriminated against in relation to their sexual orientation or gender identity (when renting; visiting leisure places, shops, and banks; or conducting financial transactions, etc.). Furthermore, Domínguez-Fuentes et al. [40] affirm that in southern Spain, 70% of a sample of 220 gay men expressed having felt rejected because of their sexual orientation. This usually involved unwanted jokes, sexual slurs, prejudiced treatment perpetrated by colleagues, abuse, obstacles to promotion in their career, or obstacles to access to work. A study carried out in Spain with 377 workers, out of which 229 were LGTBIQ+ (137 heterosexual, 134 gay, 61 lesbians, 34 bisexual), concluded that LGTBIQ+ people perceive more discrimination than heterosexuals due to their sexual orientation [10], anchoring results in data from the European Agency for Fundamental Rights–FRA [41], Di Marco et al. [42], FELGTB-COGAM [39], and DeSouza et al. [43]. Likewise, the research reveals that LGTBIQ+ people have a higher rate of work stress than heterosexuals, correlating with the studies of DeSouza et al. [43] and Badgett et al. [44], where it is clear that the stigmatizing events of the gay men’s lives were the basis for a high level of emotional stress.

LGTBIQ+ people announced higher levels of mental disorders and depression than heterosexuals [7,8,45,46]. Conversely, heterosexual women indicated a higher level than heterosexual men, but gay men presented higher levels than lesbian women, which is consistent with the findings of Balsam et al. [47], Herek [48], and Semlyen et al. [49]. Notwithstanding, as Moya and Moya-Garófano [10] substantiate, being LGTBIQ+ does not equate with low psychological wellbeing, but equates instead with discrimination based on being non-heterosexual [49]. Likewise, work stress has been related to poorer physical health [50,51], less successful professional development [52], and worse work outcomes [53]. To all of this we must add that mental disorders have an impact not only on the person who suffers them but also on the economy, work, and family environment [54].

Molero et al. [9] conducted their research with 481 gays and lesbians in Spain and concluded that subtle discrimination (such as jokes, rudeness, hostility, or unfair treatment) was superior to blatant discrimination and that the former was directly related to worse wellbeing. Thus, subtle perceived discrimination is coupled to lower self-acceptance, less control of the environment and social support, and a higher negative affect, which Schmitt et al. [55] had also discussed previously. Subtle discrimination is more difficult to detect and creates a feeling of helplessness in cases such as job search since these people do not know if they have been rejected because of their (in)ability or because of their sexual orientation. According to Molero et al. [9], Spencer and Patrick [56], or Domínguez-Fuentes et al. [40], improving social support would help prevent the negative effect of discrimination and increase the wellbeing and life satisfaction of the group. Seemingly, the passage of time also plays a consequential role in resilience and is positively connected to self-acceptance and control of the environment.

Schmidt and Kurdek [57] or Morris et al. [58] establish that the revelation and integration of sexual identity in all domains of life, including the important work environment, is one of the pinnacles of psychosocial health and psychological wellbeing in general. Work is of capital importance in life for the great amount of time we devote to it, for being normally the source of income, and for the implications it has for self-esteem, self-image, and wellbeing [10]. In this vein, Griffith and Hebl [59], Díaz et al. [60], Juster et al. [61], Rosario-Hernández et al. [62], or Rodríguez-Polo et al. [63] confirm that LGTBIQ+ people who have not openly declared their sexual orientation at work present high levels of stress and negativity in self-acceptance, autonomy, mastery of the environment, personal growth, and life purpose because the basic needs for social support and belonging to a group are not met. To this debate, it is significant to note the contributions of Rosario-Hernández et al. [62], who show that avoiding sexual orientation at work is related to depressive symptoms and, contrary to what might be thought beforehand, the fact of showing it openly is associated with anxiety symptoms.

The study by Rosario-Hernández et al. [62], on 110 LGTBIQ+ people in Puerto Rico, confirms that rather than perceived heterosexism at work, what negatively influences psychological wellbeing is actually the strategy used to manage sexual identity in it. In any case, as Borrero-Bracero [64] indicates, either openly showing sexual orientation or hiding it in the work environment is deeply affected and mediated by the type of personality, the culture, or the atmosphere of the workplace. What seems to sit at the heart of the debate, as discussed by Díaz et al. [60], is the fact of having positive built-in attitudes towards oneself as a fundamental characteristic of wellbeing, as it is the personal ability to create or choose favorable contexts that allow fulfilment of one’s own wants and needs.

#### 1.1.3. Studies on Wellbeing of LGTBIQ+ Teachers

Varghese et al. [65] specify that all elements of a teacher’s identity are involved in the teaching and learning process. For this reason, it is essential to understand what happens in the classroom and in the educational ecological system, as a critical sociocultural and political component, and to determine the parameters under which the teaching of a language is established. This idea coincides with that of Clarke [66] when he affirms that our ways of being teachers are shaped by our ways of being people. Along this line, Nelson [12] plugged into the impossibility of separating sexual identity from other facets of teacher identity. Examined through a post-structuralist lens, it is a multidimensional cultural construct that is considered to be dynamic, diverse, and subject to space–time precepts [67].

For Varghese et al. [65], the identity of language teachers is composed of three levels that interact in the same system. At first, they speak of the inherent instability in which the teachers’ own determination is essential for aligning their identity with the guidelines of the educational context. Another relevant factor would be the political, social, and cultural context, with its specific role in the formation of the teacher’s identity and the adjustment dialogue that is established between the contribution of the self and the external influences. Lastly, the role of language and discourse seems key when it comes to constructing and negotiating identity. In this sense and with regard to sexual identity, Nelson [68] points to the fact of social and discursive interaction as a producer and interpreter of sexual identities.

Despite all the legislative advances in the Western world, there are numerous studies that confirm that LGTBIQ+ teachers continue to suffer discrimination in the educational environment [1,5,6,69,70]. Thus, heteronormativity permeates the entire school and educational culture, from the subjects themselves, to the educational policies, the curriculum, and the classroom discourses. Anyone who tries to get away from it is punished with silencing and invisibility, as evinced by Foucault [71]. Bearing in mind these premises, we could state that educational institutions continue to be conservative, and they act as supervisors of the private and work identity of queer teachers today.

Ferfolja and Stavrou [13] contend that this discrimination pertains to silencing in terms of psychosocial and personal wellbeing, as well as pertaining to costs in the workplace, productivity, and the establishment of an LGTBIQ+ friendly culture in schools. They [13] suggest that the effects of discrimination seriously affect identity, relationships, physical and emotional health, personal growth, career opportunities, and job retention. These actions are consistent with the studies by Irwin [72,73], who also mentions physical and sexual assaults, damage to private property, and ridicule through jokes or humiliating actions. Jones, Gray, and Harris [74], and Ferfolja [75,76,77,78] have denounced the idea that the sexuality of teachers still remains hidden from their students and that teachers do not know the legal and political protections in terms of discrimination in the work area. DeJean [79] brings to the fore anxiety as an effect of the concealment of sexual subjectivities, which is anticipated as an element that erodes wellbeing.

Ferfolja and Stavrou’s [13] study of 160 gay and lesbian teachers in Australia reveals an invisibility policy, meaning that “many teachers, including those who wish to be proactive about these issues, have little guidance or support from the schooling institution” (p. 123). It is perceived that dealing with homophobia can lead to problems with students, colleagues, managers, or families. This research brings to light the idea that educating students in sexual diversity is a relevant factor in developing a school culture where LGTBIQ+ teachers can feel comfortable and be who they are. These data correlate with findings from Gray [16], who demonstrated that teachers who disclose their sexual orientation in their workplace and talk openly about their sexuality and relationships have a greater feeling of belonging to the community and report higher levels of job satisfaction and a healthier connection between their private and public-professional “selves”.

Moreover, in the Australian context, research by Ullman and Smith [14] with 1036 teachers from New South Wales indicates that 43% had experienced discrimination, abuse, or disadvantage because of their sexual orientation. The authors focused on verbal or psychological bias on the part of colleagues and students. In addition, participants declared having felt inspected regarding employment opportunities by the management teams of their schools. The principal findings of this research are that many teachers perceived their psychological wellbeing as being diminished as a consequence of the discrimination mainly suffered from the management teams and that they needed external help to be able to advance. It is surprising that only 8% of the discriminated reported the situation to their superiors and were satisfied with the answers they obtained. Although the effects on the self-efficacy of these teachers were not significant, those teachers who participated openly in support measures and visibility of their LGTBIQ+ identities proclaimed greater wellbeing and a sense of belonging, credibility, and trust in their schools and classrooms. Lines of action were extracted from the study, such as the need to educate about gender diversity and sexuality in schools to promote teacher wellbeing; the need to implement clear and precise anti-discrimination measures; and the need to train management teams in sexual identities so that teachers could feel safe and boost their wellbeing.

The debate on how LGTBIQ+ teachers handle their sexual identities at work seems to diverge from the data we have so far and, above all, seems highly dependent on the cultural and temporal context. In Griffin’s seminal work [80], different strategies were established such as “passing”, “covering”, or being out of the closet in an implicit or explicit way, where everything was context-bound. Similarly, Nelson [68] floats the idea that queer language teachers are forced to choose whether or not to come out of the closet in their professional context. Many think it is essential, while others prefer to skirt problems [6]. Ferfolja [75,76,77,78] makes explicit that these issues will always depend on the different environments and that not all teachers feel discriminated against. However, those who experience discrimination are not always conceived as victims, but rather as agents empowered to correctly navigate the discriminatory context.

In the British context, Edwards et al. [81] suggest that queer teachers consider it awkward to come out of the closet in the workplace, especially in the early years of their career. This perception contrasts with the opinions of LGTBIQ+ Spanish secondary school teachers who decide to openly show their sexuality in the classroom because they say that it is a pedagogical position that helps to dispel prejudices and strive against homophobia [82]. It is also their contention that it helps adolescent students to grow up with role models, although it is not easy due to the pervading homophobia in schools. Teachers are still afraid of families’ and students’ reactions, harassment, and insults. They agree on the idea that silence and invisibility are capable of maintaining homophobia and that the more it is addressed in the classroom, the more homophobia will decrease. In the homophobic imagination, the pervasive idea that equates homosexuality with pedophilia continues to exist. Consequently, teachers may not dare to come out lest someone should think ill of them [69].

In Spain, transsexual teachers are deeply involved and decide to come out in Secondary Education to stand up to bullying and create safe spaces for all students. Some teachers, such as Víctor, a trans music teacher, said: “If I had had a trans role-model, to be able to see him/her and admire him/her... I would have said it was not so weird. Now I can help adolescents a lot”, or “Adolescence for a trans person is very complex because you are in no man’s land, you do not belong to one side or another. I felt very lost and I felt very lonely, everything was very difficult. I turned to music and there I earned the respect of the people, music saved my life” [82]. From this teacher perspective, it is acknowledged that self-esteem and valuing oneself are key to fighting against LGTBIQ+ phobia. This coincides with Lander [5], who stated that “learning to teach is about…negotiating identities, finding out who one is in the classroom” (p. 75).

Studies on the wellbeing of queer university foreign language teachers are virtually non-existent, and we can only point to Lander [5] and Lin, Trakulkasemsuk, and Zilli [6] as related works. Lander’s qualitative research [5], with three university professors of English as a foreign language in Colombia, obtains threefold conclusions: (1) the array of personal experiences is so complex and varied that we cannot reach universal conclusions; (2) queer identity and the identity of language teachers are not incompatible with wellbeing, despite Colombia being a conservative country; (3) the religious persuasion of the institutions does not seem to be the problem that erodes teacher wellbeing, but rather the specific and non-transferable attitudes of particular individuals within the institution. In any case, the immediate professional context does draw a high-impact picture for the wellbeing of these teachers. Lander’s [5] work confirms what Nelson [12] pointed out in relation to the fact that all teachers, queer or not, bring the whole range of their identity to the classroom and that the sexual orientation trait, for example, cannot be dissected easily. In this case, not hiding it promotes the dynamics of the classroom and helps the learning process.

Moreover, the qualitative study carried out by Lin, Trakulkasemsuk, and Zilli [6] with a professor of English as a foreign language at a university in Thailand sheds light on interesting elements of how sexuality is negotiated in the process of interaction with different agents. The study discloses the importance of the fluidity of queer subjectivity through the different discourses and how teacher self-imposed strategies are used to negotiate the boundaries between the queer and the professional self. These readjustments suggest that the queer teacher is mainly a foreign language professional, once their sexual orientation has been normalized in the educational context.

### 1.2. Focus of the Work and Research Questions

The objective of this research was to examine under the ecological paradigm [18] the teacher wellbeing of queer foreign language university teachers in a Spanish context.

Consequently, the research question that anchored this study was the following:

What are the links and the interplay, following an ecological model, of sexual orientation and the participants’ wellbeing?

## 2. Materials and Methods

### 2.1. Context

The research was carried out in the region of Andalusia (Spain) in the first semester of 2021. In Andalusia there are 9 public universities that offer language programs in teaching degrees—namely, University of Jaén, University of Córdoba, University of Seville, University of Pablo de Olavide, University of Huelva, University of Cádiz, University of Málaga, University of Granada, and University of Almería.

### 2.2. Informants

In order to attain the objectives, we selected the informants following a judgmental or purposive sampling [83]. We sent an email to university teacher training departments in the region of Andalucía (Spain) seeking queer male foreign language teachers who might be interested in participating in this research. We received 10 answers, but finally only 7 of them accepted to be interviewed on the condition of anonymity (1 from the University of Jaén; 1 from the University of Córdoba; 1 from the University of Seville; 1 from the University of Cádiz; 1 from the University of Málaga; and 2 from the University of Granada).

The main sociodemographic data of the informants are portrayed in Table 1.

### 2.3. Instruments

In order to collect the data in the utmost comprehensive manner, we designed in-depth interviews with open questions. We considered that the interview was the instrument that would better adapt to the profound qualitative information we sought to obtain from the participants. An extant literature review guided this process of setting questions and variables of common use in this field of knowledge, with special focus on the works of Ryff [84] and Ryff and Keyes [85].

Table 2 summarizes the main thematic areas covered in the interviews:

Once the interviews were designed, they were validated by three experts in educational qualitative research. In this process of external evaluation, the experts suggested thematic changes, improvements in the writing of the questions, and better adjustment to the research question and the objective of the study. Changes were discussed thoroughly with the external evaluators before producing the final validated instrument.

### 2.4. Practical Implications, Research Ethics

With the aim of protecting the informants to the maximum, we followed the accepted procedure for ethics and good practices in research (in compliance with the Declaration of Helsinki and the Ethics Committee of the University of Granada, Spain, protocol code 2006/CEIH/202). The informants received a written letter explaining the rationale of the research and the detailed description of the whole process of investigation. Additionally, every person was informed orally of this protocol; it was made explicit that their participation was voluntary and that their privacy would be totally preserved. In the same way, the informants had the liberty of abandoning the research process at any time and data collected so far would not be considered.

### 2.5. Process

The informants were contacted in January 2021 and later interviewed during February and March 2021 using Google Meet (the COVID-19 pandemic situation prevented us from interviewing them in person). Two members of the team interviewed all the participants since they had previous experience in this type of research.

The interviews were taped by enabling the recording option in Google Meet. Each interview lasted between 50 and 90 min. Later, they were transcribed to paper to process the data in a faster way. In the resulting text (a corpus of 53,221 words) the data were effectively marked with signs to discriminate discourse features of rhythm (+, ±, −), tone (√, ~, x), emphasis (!), pausing (//) and time (…). Interviews were carried out in Spanish and later translated into English. To ensure data accuracy, English translations from the original Spanish transcriptions were carefully reread and proofread. This task was undertaken by a researcher of this study who is a professional translator.

### 2.6. Method for the Analysis of Data

Having followed Bronfenbrenner’s [18] ecological paradigm for the study of teacher wellbeing, the classification and analysis of data adhered to the 5 sub-systems: microsystem, mesosystem, exosystem, macrosystem, and chronosystem.

We want to make it clear that data related to the psychological capital of the participants were analyzed in a transversal manner throughout all the sub-systems, with special focus on the micro-level. Jin et al. [4] have agreed the microsystem to be the lynchpin of all the identity factors and personal and professional competences. However, all the sub-systems form an interwoven network, and most variables travel functionally through them.

This was a qualitative study through which we pursued a deep understanding of the wellbeing of queer male university teachers. From our prior forays into qualitative research, we adhered to the tenets of content analysis [86,87,88]. This has been defined by many authors since it reached wide consensus in the 1950s in the United States. However, we focused on interpretations by Weber [86], Krippendorff [89], and Cohen et al. [90]. The former notes that “content analysis is a research method that uses a set of procedures to make valid inferences from text” [86] (p. 117), while the latter expounds that it is “a strict and systematic set of procedures for rigorous analysis, examination and verification of the contents of written data” [90] (p. 475). Krippendorff’s [89] definition adds the nuance of replication—“a research technique for making replicable and valid inferences from texts (or other meaningful matter) to the contexts of their use” (p. 18). In general terms, the research method is a multitiered process that goes from codification, categorization, and comparison to the establishment of conclusions from the data of oral or written texts. This allows the encapsulation of great amounts of raw information with the aim of engendering meaning that is trustworthy and rigorous [86].

We want to make explicit that the whole process of data analysis in qualitative research is long, complex, and sometimes frustrating. As such, the analysis was transversal to the process from the very beginning to the end when listening to the recorded interviews, transcribing them, summarizing the content to establish matrixes, coding information, translating data from Spanish into English, synthetizing categories according to the research question, finding connecting or divergent topics, selecting quotations, or writing the final report to explain the meaning in a coherent manner.

Since we had a manageable number of interviews, we decided to conduct a manually analytical process, and no use of specific research software was made. Once the textual corpus of the study was defined after the transcription of the recorded interviews, we proceeded to the analysis of the data based on the classical phases of content analysis [83,91]: decontextualization, recontextualization, categorization, and compilation. This allowed us to delve into a level of latent and interpretive analysis of the deep and underlying meaning of the data [92].

### 2.7. Validity and Reliability

Following Catanzaro [93], to obtain a critical view as researchers and as a measure to increase the validity of the data, we asked an independent researcher specialist in qualitative research to read the resulting text and to judge whether or not it was reasonable. His evaluation was positive, and the coherence between the matrix corpus and the scientific analysis accomplished was verified.

The fact of interviewing seven foreign language teacher trainers (specialists in English, French, and Spanish) of different universities, ages, and cultural contexts provided sound elements to support the validity and reliability of the study, with views to the triangulation of data. Their varied perspectives on the interrelation of sexual orientation and wellbeing offered a well-balanced scaffolding to become cognizant of the inner architecture of teacher wellbeing.

With the purpose of increasing the validity of the study, the four researchers performed a profound data analysis individually. Later, the results were shared and discussed to reach a consensus about them. Thus, we made sure that they indubitably reflected the phenomenon studied. In the same way, if this corpus of study were to be analyzed by other researchers, the results would be the same.

## 3. Results

In order to facilitate the reading and understanding of the results of this study, we decided to organize them according to the research question posed at the beginning of the study, taking into account the ecological model of Bronfenbrenner [18]. Therefore, along the following lines, we address the interplay of sexual orientation and the personal and professional factors that influence the teacher wellbeing of the target cohort in relation to the micro-, meso-, exo-, macro-, and chronosystem.

### 3.1. Research Question: What Are the Links and the Interplay, Following an Ecological Model, of Sexual Orientation and the Participants’ Wellbeing?

First, we present Table 3, which showcases the main themes that emerged in the interviews and that allow us to clearly understand the complex data system and interconnection between sexual orientation and the rest of sub-structures of teacher wellbeing as an ecological paradigm.

#### 3.1.1. Microsystem

(a) Self-concept as LGTBIQ+ person

Regarding the self-concept as LGTBIQ+ people, most of the participants had a high, positive assessment of themselves, with some of them explicitly indicating that being queer is a natural condition that one cannot choose:

*When I found out that I was gay [//] I knew in advance that it was inside my head [//] even if I didn’t know what it was for a long period. I really think that sexual orientation is not chosen [√]. I have not chosen to be gay, but I have found out that I am gay. So I am what I am, and I am very satisfied [!]. The most important thing is not finding out what your sexual orientation is but to know who you are [!].*

*(P7)*

This positive self-concept is essential for their psychological and educational wellbeing. For P1, being openly gay is an element of liberation and empowerment, explicitly stating that he could not live at the best of his potential until he accepted his sexual orientation. In this sense, they feel the support of the existing regulations in Spain that are explained in the macrosystem below. However, P4 does state that until the age of 30, he wished he were not gay in order not to have suffered the plight he experienced in his childhood, adolescence, and early youth.

Likewise, all the participants are currently satisfied with their biological sex as men and they also accept many of the sexual identity features traditionally considered as feminine in their culture.

It is worth mentioning that P5 added that the fact of defining, naming, and classifying sexual identities can limit, stereotype, or pigeonhole people and restrict all the other things they could be.

(b) Sexual orientation in the identity spectrum

All the participants declared the importance of the intersectionality of sexual orientation with other variables of people’s identity. P4 and P5 made explicit the idea of decentralizing sexual orientation across the spectrum of a person’s identity. They complained about the fact that many LGTBIQ+ people hyper-identify themselves with their sexual orientation and forget about the complex, multifactorial, and multivariate myriad of the identity spectrum through time and space. This contrasted with P1 and P7 who felt and behaved in a strong homosexual-centered manner.

P2 and P5 claimed that discrimination against LGTBIQ+ people often derives from other identity areas, such as class, race, educational level, employment or gender, and not because of sexual orientation in itself. This is mainly related to the idea of occupying power positions in society, i.e., centrality or marginality. P7’s words better illustrate this idea:

*I am an activist person [!], my husband and I, and my family in general, are activists. So, for us, there is a fundamental idea: please stop discriminating against people because of their sexual orientation [...] I think that we fight for the respect of human rights in general, which includes sexual orientation and the rest of the problems that people have due to religions, politics[+], and so on [...] I am always very attentive to the achievements and the laws that are enacted for the recognition of LGTBIQ+ [...] I am gay, so I don’t want to hide it [√], I don’t have to hide it, I don’t have to wear a sticker that says I’m gay, but I don’t want to ignore it at any time [!].*

*(P7)*

P5 stated that the labelling of sexual identities normally acts as cultural hindrance rather than as acknowledgment of opportunities. Along the same line, P6 would prefer to erase the layers of sexual identity when approaching people and perceive them on other bases—namely, their intellect, cultural level, or life experiences. However, P1 saw these themes as political opportunities for change, for activism, and for the betterment of the world. For him, being gay is essential to his identity as a teacher and teacher trainer since he can explore untrodden paths towards social justice.

(c) Self-commitment

P1 reported that being gay implies for him a greater self-demand when it comes to functioning socially. He felt that he had to continually prove that he can do things the same as or better than straight people, which was very stressful for him. The rest of the participants did not share this perception, although P4 observed that some people have demanded more of him (either in the private or the professional sphere) based on his sexual orientation. This fact deeply outrages him, and he considers it a very powerful form of subtle discrimination.

*I think I sign up for any university committee to show people that a homosexual person can do the same job as a heterosexual person [√, ±, !].*

*(P1)*

(d) LGTBIQ+ models of identification

All the participants, except for P1, declared that they had not had role models of LGTBIQ+ people in the educational field and that this has impacted negatively on their self-assertion. On his part, P1 pointed out that a great role model for him was his thesis supervisor, who is openly gay. He helped him gain self-confidence and showed him that he can do everything in his academic career without fear of being discriminated against for his sexual orientation. P3 explained this question by saying that he has had LGTBIQ+ models, yet he never gave relevance to them as queer people, but rather for their ethical or professional values.

In the private sphere, it is worth mentioning the contributions of P1, P5, P6, and P7. For P1, his models have been his sentimental partner, with a great talent for teaching, celebrities from the drag world such as RuPaul or Bianca del Rio, and singers such as Lady Gaga or Cher. From all of them he learned the lesson of the importance of first loving himself in order to be able to love another person in a healthy way. He has experienced their influence as subconscious life-coaches and courageous models. P5, for example, highlights the iconic role of Spanish folk divas for gays, focusing on such prominent figures as Lola Flores or Rocío Jurado. All these Spanish show-women are artists who fully embraced the rationalization of the same-sex cause and espoused gay-friendly stances in public. Additionally, their flamboyant way of being, their great talent as singers of melodramatic songs, their capacity as actresses, or their way of dressing and behaving, allowed them to dream and escape from a black-and-white world. They all brought color and fantasy and allowed gay men to fictionalize their lives with music and boundless femininity, i.e., a whole antagonistic world to the cisheteropatriarcal masculinity they were intended to perform. P6 added that he prefers not to have LGTBIQ+ models because, according to his experience, a high percentage of this group contribute to his own ghettoization, and his beliefs are deeply contrary to their way of understanding sexuality as performance. P7 said that he has had many models of LGTBIQ+ people in his context, but that none have been sufficiently important to serve as personal or professional references. P2 reported linguistic anxiety for not having had models of openly French LGTBIQ+ speakers with whom to identify himself.

*I think that not having openly LGTBIQ+ speaking models during my learning of French has made it difficult and anxious for me to identify myself in the other culture [!], which also affects me on a linguistic level [//].*

*(P2)*

(e) Personal development

The interaction between sexual orientation and personal growth was tackled from different angles. For P1, P2, P5, P6, and P7, sexual orientation functions as a powerful motor for personal growth that, heterosexuality, on the contrary, is not able to accomplish. It is the need for navigating the conflict and the margins that educates queer people and makes them grow, despite the hard and negative conflicts. In this sense, P5 declared that sexual orientation causes a plethora of problems that impinge negatively upon people’s psychological and emotional wellbeing, but that it can also be an active tool for struggle and growth and not only for generating a breeding ground for victimization and stagnation. However, for P3 and P4, sexual orientation does not represent a stimulus to continue growing, at least with the same strength that other components of identity may exert in their lives.

(f) Lifelong objectives

Regarding the role of sexual orientation in life goals (a very powerful indicator of psychological wellbeing) only P7 declared that it has had an especially relevant role through the adoption of a daughter together with his partner. He is fully satisfied with the way his life has turned out mainly because of his continuous commitment to make her happy, demand a fairer world for her, and being able to grow old with his partner watching her daughter’ progress in life.

(g) Influence on students in their role as LGTBIQ+ teachers

In the context of the classroom and by virtue of the influence that these teachers declare to exert on their students in relation to sexual identity, we highlight several elements of paramount importance that greatly favor teacher wellbeing. On the one hand, it stands out in the perception of all, except for P3, their role in serving as LGTBIQ+ models for their students. This demonstration in university classrooms leads many queer students to ask for their advice and support because they come from oppressive rural contexts or, religiously speaking, ultra-conservative families. Other queer students, shy by nature, have participated more in class when they became aware that they were being taught by queer teachers (P4). They expressed their heartfelt gratitude for his inclusive policies.

Regarding the teaching methodology, P1, P2, P4, P5, and P7 have adapted or modified certain content in their courses to introduce a queer perspective and address issues such as homosexual marriage, respect for LGTBIQ+ human rights, representation of queer people in the media, offensive language, or creative fictional uses of literature. They all spotlight the honesty involved in being openly queer, serving as a model of freedom of expression and critical thinking as a useful means to fight against oppressive cisheteropatriarchal structures.

*As a teacher, I teach them lots of taboo subjects and how to cope with them in primary and secondary schools from a positive, natural approach [...] So when you talk to them about the pedagogy of affirmation, queer theory, gender theory, sexual affective diversity, for example [//], or even death [...] I think you leave behind the well-established archetype of a traditional teacher [...] My students really appreciate it [!].*

*(P1)*

(h) Dealing with LGTBIQ+ issues in the classroom

The student body, according to all the participants, is the great support of LGTBIQ+ issues in the classroom since the majority of them are very openminded and tolerant. Moreover, great strides have been made in the Spanish educational landscape in this direction. However, P7 reported that he often has problems with US students when dealing with sexuality issues in the classroom because in their cultural context they are not used to it, as well as when sexual orientation intersects with religion or politics. However, this teacher makes it clear that positive personal relationships with most of his students are one of the main sources of motivation and professional wellbeing today.

For his part, P5 commented on the case of a student who boycotted his class and accused him of indoctrination when dealing with issues of representation of sexual identities in the media. This teacher’s response consisted of listening to her arguments and putting reasoning, the use of democratic procedures, and critical thinking before subjective opinions. In the end, the student understood the pedagogical stance, and he also felt supported by the rest of the class. Although everything ended up well, this case caused him a lot of emotional stress and loss of confidence and self-esteem in his work setting.

P2 reported that for a few months he worked as a secondary school teacher in the southeast of Spain and continually hid his sexual orientation in the classroom for fear of being attacked by students or families.

All the participants, except P3, pointed to the prevailing need of teachers from university classrooms for initial and continuous training in regional educational administration policies. They claimed that the focus should move from the LGTBIQ+ community (i.e., the victims) to the violent social structures and the aggressors.

#### 3.1.2. Mesosystem

(a) Influence that other people exert on participants’ lives

Regarding the relationship of sexual orientation and the influence that other people exert on the participants, all of them agreed on the great impact the opinion of others has had in their lives. Effects were more evident during their adolescence because their autonomy was reduced, and consequently, their psychological wellbeing lessened. P7 recognized that in his adolescence, his environment exerted a lot of pressure on him, but this situation changed when he traveled abroad to France and Italy, became independent, and established a formal relationship with his current partner.

However, all declared that the passing of time, social learning, and maturity have served to reduce this influence and they have been able to achieve a certain balance in their autonomy in terms of interpersonal relationships.

(b) Expressing opinion in public

Sexual orientation interfered when expressing opinions in a group only in the case of P1. He indicated that he feels shy to express what he believes or feels in private or work meetings if there are a majority of heterosexual men. Nevertheless, if the interlocutors are women or LGTBIQ+ people, he feels free and manages to express himself autonomously. In addition, he affirmed that to avoid conflict he prefers not to surround himself with people, friends or colleagues who do not think like him, which has turned out to be a noteworthy obstacle to his wellbeing. For him and the rest of participants this condition has not undergone variations over time.

(c) Impact of sexual orientation in private relations

Regarding the implications of sexual orientation at the private level, we must highlight aspects such as: the sense of non-belonging or exclusion from social groups (P1), the feeling of being constantly under suspicion (P5), and the need to freely express their sexual orientation in any context of the private sphere in order to promote adequate social interaction and develop psychological balance (P2, P4, P6, P7).

In this respect, P7 declared that he received insults from his ex-brother-in-law when he found out that he was gay. Additionally, the same girlfriend, in revenge, informed his entire context of his sexuality, with the subsequent psychological damage that this caused to him. This happened in the 1980s in an oppressive small village in the north of Spain.

(d) Impact of sexual orientation on school life

All the participants commented that they were victims of homophobic harassment during the years of Primary and Secondary School (1980s and 1990s of the 20th century in Spain), harassment that P7 even experienced during his in-placement practice period at university. The nature of harassment was usually verbal, with insults, belittling, and ostracism. P1 and P7 added that teachers witnessed this harassment and did nothing, thus becoming accomplices of the bullies, showing total incomprehension and indifference. This harassment, according to all the participants, has had deleterious consequences that have eroded their psychological wellbeing, such as fear, insecurity, anxiety, stress, addictions (alcohol and marijuana consumption), depression, low self-esteem, lack of self-control, or living sexual relations with taboos and frustration. P1 self-harmed when he suffered homophobic bullying at school as a child and wanted at that time to change gender in order to fit into the cisheteropatriarchal system. P2 declared that he suffers a lot of emotional stress as he is continually forced to come out in front of every new person he meets in life, privately and professionally. That is, for him to align beliefs and actions leads to psychological stress.

*I come to the conclusion that your sexual orientation should not mean constantly leaving your comfort zone [...] going out of the comfort zone is positive [~], but when it has to be done continuously [x], obviously it has a price [√], and then you pay for it with your mental health [...] if you manage it well, then you manage to grow as a person [~], but there are times when this departure from the comfort zone can be abusive [!], can’t it?*

*(P2)*

However, all the participants agreed on the fact that they positively value how the passing of time and the continuous feeling of struggle and improvement has helped them to handle all these problems in a satisfactory way—to the point of being able to affirm that they currently enjoy a state of acceptable-positive health.

In most cases, it has been a process of self-learning and self-care, with the exception of P5 and P6 who have needed pharmacological treatments and psychological therapy to be able to overcome these problems. In any case, they added that they must be constantly on the alert because mental illnesses or emotional issues always loom large and are liable to reappear under stressful conditions.

Hence, dealing with these situations has been a driver for personal growth and improvement (P1, P2, P4, P5, P6, and P7). In other cases, it has given them a solid reason to become activists for LGTBIQ+ rights (P1, P4, P5, P7).

(e) Impact of sexual orientation on family life

The family environment has also been a social space full of stressors for the wellbeing of this cohort. All participants declared to have had conflicts in the family environment of varying nature. P1 said that his maternal family did not accept his homosexuality in a southern rural area of Spain. The rural environment of childhood was also declared to be hostile by P4 and P5, where homosexuality was conceived as a contagious disease, stigma, or social shame. P2 added that his ultra-Catholic family associated homosexuality with HIV and mental illness, also corroborated by P5, his family being agnostic-atheist. P5 explained that although he has not been explicitly discriminated against by his family, he continuously perceived that he did not meet the expectations set by his parents in terms of gender roles and sexual orientation. P7 went so far as to affirm that he had to leave his home because his father and brother ignored him, a fact that coincided with P5 on the part of his mother and sister.

This hostile climate in the childhood and adolescence of the participants has turned into a more benevolent climate in adulthood. For example, P1, P4, and P5 declared that this is all an education problem and that they are also there to educate their families. Likewise, all the participants agreed, in relation to their families, that the focus should be on the cisheteropatriarchal structures that make violence based on sexual orientation possible, rather than on the specific people who reproduce those discourses and actions they learned from the socially prevailing system. P2 added that these imbalances in family relationships have a high impact on satisfaction with social relationships in general, both in the private sphere and in the workplace, one of the backbones of psychological and educational wellbeing.

(f) Perspectives on LGTBIQ+ people

All the participants, except P3, stated that they are very conscientious to avoid injustices and violence against LGTBIQ+ people. They all consider that a life objective is the fight against the cisheteropatriarchal system, either in local, national, or international contexts. They especially emphasized how dangerous the world is, outside the Western world, for people who sexually deviate from this model of cultural construction of sexual identity. This life purpose and way of understanding the LGTBIQ+ community serves as a balancing act for teacher’s wellbeing, which is prone to be successfully developed within their profession.

(g) Interplay of sexual orientation and university life

The implications of sexual orientation in the universities of our participants are addressed by considering variables such as management teams, colleagues, students, research, and knowledge transfer. P3 veered away from the rest of interviewees when he stated that he has never had any explicit problem related to his sexual orientation, since he conceals it as it is a private issue that nobody should know about. The differing position of this participant may be due to his country of origin, in the Balkans, where he lived until the age of 25. This is a well-known area of homophobic tradition with a high prevalence of the unwritten law “don’t ask, don’t tell.”

- Management teams: P5 and P6 reported problems with the management teams. P6 informed that in the past he had serious troubles with the management team of his former private university. They even had to go to court, which caused him high stress levels. In his current public university, he does not have these kinds of issues, but he has the perception that they could arise at any given time.

P5 suffered homophobic harassment by the head of his department at the beginning of his academic career when he faced job instability. He received personal insults, humiliations, invitations to leave the job, yelling, public disparagement of his CV in front of other colleagues, obstacles in job promotion, and even accusations of wanting to sexually abuse students. This participant, in trying to explain what happened in the past, pointed to professional envy for introducing new ideas and an insane understanding of competitiveness. He also heralded the personal and sexual frustrations of the bullies and the university cisheteropatriarchal structures, which remain veiled and unquestioned. Another factor was the cowardice of aggressors who chose to victimize people under job insecurity, since he never saw full professors being attacked for these reasons. This amalgam of elements generated mental illness, such as nervous depression and bulimia that needed drug treatment and psychiatric therapy over time, turning into a chronic problem. He took refuge in food to counter this situation and gained up to 40 kg.

*[…] I remember that the head of the department of my university at that time called me into her office one day and began to insult me [x] and tell me all kinds of questions related to my personal and professional image, and I left her office crying [x]. It was a really difficult episode, an episode that gave me [//] a mental illness [-], which would be a nervous depression from which I had to spend many years in psychiatric treatment [//] after many years I managed to overcome it [~], but when one has this type of issues, one is like a permanent sick person [√], we could say a chronic patient: you learn to handle the disease itself, but you always have to be alert because any stressful situation or any problem that work, life, or whatever may cause you, it could make you suffer a relapse [‒].*

*(P5)*

This whole situation triggered a learning process, and the initial victimization gave way to an opportunity for activism and reorientation of teaching and research for LGTBIQ+ social justice. Of special relevance in this sense is the standpoint of P2, P4, P5, and P6 with regard to resilience. Although it helps to move forward and manage in the environment, most of the times it favors the educational institution and violent oppressors more than the teachers themselves. They must navigate a tortuous path of pain until they manage to heal themselves almost without any help from the system.

- Colleagues: P2 perceived that his sexual orientation was an issue that bothered his colleagues, even if they did not say anything about it. In fact, he targeted fellow teachers who avoided his friendship for being gay. P1 was the subject of homophobic jokes and P4 felt judged and discriminated against by conservative and highly competitive colleagues. P6 said that his colleagues did not publicly support him in his problems with the management team and P5 only felt supported by a small group of fellow friends, while a large number of them positioned themselves next to the head of the department and encouraged her to continue harassing him.

P4 and P5 also declared that they have had serious and uncomfortable problems in evaluation panels of MA dissertations in the face of homotransphobic comments from colleagues. This is an issue that generates a lot of stress because many students want to address LGTBIQ+ issues in their foreign language projects, and there is latent fear that the examining boards will attack them on that matter.

- Research and knowledge transfer: At the research level, P4 and P5 reported a series of events that greatly undermined their teacher wellbeing from perceived homophobia in the processes of publication or presentation of papers on LGTBIQ+ topics in foreign language conferences of Spanish professional associations belonging to the applied linguistics domain. For example, they received the rejection of a communication proposal to a conference, arguing that it was a topic more typical of cafeteria conversation than of professional study. The same happened with other prestigious international organizations and journals in the United States. Along another line, one of the participants, P1, values very positively the opportunity that his university has given him to establish relationships and transfer of knowledge between university research and LGTBIQ+ associations in his workplace.

#### 3.1.3. Macrosystem

(a) LGTBIQ+ legislation in Spain

All the participants felt proud and highly valued the existing LGTBIQ+ legislation in Spain and considered it a pioneering country in the entire world (except for P3, who said that he did not know anything about politics or legislation). However, they are afraid of the rise of far-right parties in Spain since they could abolish or jeopardize these legislative advances. In fact, in the regions they are governing, they have implemented the so-called “parental pin”, by virtue of which families have the power to veto teachers from dealing with LGTBIQ+ issues in their classrooms. This is a very serious fact that goes against basic democratic principles, as for example the academic freedom protected by the Spanish Constitution of 1978.

What worries them most in this sense, as teacher educators, is that the law is more advanced than the mentality of the Spanish culture, which is still awakening after forty years of Franco’s dictatorship, which was of an inquisitorial nature for LGTBIQ+ people, considering them sick, degenerate, and lazy and to be crooks.

It also engenders concern in all the participants to see how in the educational sphere both in Primary and Secondary Education the law is not fully applied, as if it were only a whim of women and LGTBIQ+ people and had nothing to do with human rights (P1, P2, P4, P5, P7).

*As a same-sex family [!], we are forced to constantly demand that my daughter’s school address LGTBIQ+ issues of core relevance for the acceptance of family diversity [±].*

*(P7)*

They also pointed to the shortage of appropriate teaching materials or good teaching practices in this regard. The lack of initial and continuous training of teachers was also denounced. Additionally, they declared to having been blamed, accused of pedophilia by families, or questioned by families and management teams for lack of professionalism (P1, P2, P4, P5, P6, P7). In short, although the law is progressive in scope, it is not enough to fight against LGTBIQ+ phobia in society, unless it is accompanied by a rigorous and effective educational plan dependent on public administration.

(b) Interplay of sexual orientation and the area of foreign languages

The interrelation of sexual orientation with being foreign language specialists was almost unanimously positive and favored their psychological and teacher wellbeing. Foreign language, as an academic area, has been for everyone, except for P3, a way to openness and tolerance towards otherness. The fact of traveling abroad to European and Anglo-Saxon countries has helped them to accept themselves and, above all, to escape the oppressive contexts they lived in during their youth in Spain. For example, P5 positively values the Anglo-Saxon world for representing innovation and modernity and allowing him to be in continuous contact with socially challenging theories such as the Crip Theory that intersects queer sexuality with able-bodiedness. In addition, feeling the “others” in other cultural contexts has enabled them to have a critical and reflective lens on their own cultures and ways of understanding sexual identities. All this has helped them, as stated by P1, P2, P4, P5, P6, and P7, to assert themselves and, ultimately, to flourish personally and professionally to the maximum of their potential.

*Languages have opened my mind [...] when you live in another country and when you have contact with other societies, you realize of many things that shape your identity such as their ethical, moral, religious, political values [±]. So, learning a language offers you a passport to discover and metabolize many other cultures. This has a lot to do with the way you teach a Foreign Language [!].*

*(P7)*

#### 3.1.4. Chronosystem

As explained above, we must reiterate that the passing of time, together with personal learning and self-care in conflicts, have allowed the participants to reach a wellbeing state that can be envisaged as stable. This means that teacher wellbeing is a multilayered psycho-social construct that evolves through space and time in its complexity of variables, menaces, and perspectives.

*As we go back in time, there were more problems regarding sexual orientation, which had a greater impact on my personal or emotional development [x]. However, as you mature, you learn how to relativize this issue much more [~], and you care less about the opinion of others [~].*

*(P4)*

Therefore, the participants’ self-identity construction process relied fundamentally on their assumption or rejection of their sexual orientation across time, as it represents a cultural form of social exclusion that has been exerted against them even before they knew they were gay or bisexual. Consequently, this construction process is strongly influenced by the multiple and diverse discrimination episodes they have suffered in their social and work environments.

*I was called faggot, sissy or swishy, and some other offensive words, several times when I was just a kid [+]. I didn’t even know what it meant at that time. This type of harassment got worse when I was a teenager. However, isolation or just the feeling of not belonging [//] hurt me even more than insults [±].*

*(P1)*

*When you are a child, other children and even adults use your yet undefined or undiscovered sexual orientation to exercise influence over you in different ways [±]. I think that the fear to be gay is used as a patriarchal form of power […] I feel that some of my colleagues, especially those who are more conservative, prefer not to collaborate with me [//] or try to avoid telling me some important information just because of my sexual orientation [-].*

*(P2)*

In parallel, this process is also shaped by some strategies they use to cope with those episodes of discrimination, such as the use of resilience strategies, LGTBIQ+ activism, and pedagogy of affirmation. This learning process has a positive impact on their identity construction and their current wellbeing.

*Learning and teaching a foreign language strengthens your capacity to adapt thanks to resilience [-]. This means that learning a language implies making mistakes and knowing how to overcome them. But learning and teaching a language has a lot to do with otherness too [~], so it helps you not only finding and assuming who you are [!], but understanding other people or cultures, which has a positive influence on your daily relationships [±].*

*(P5)*

*Being an openly gay teacher makes me feel good on my skin [√]. I feel I am creating a more suitable future for other people that have suffered from similar or just the same type of discrimination […] Affirming myself in class [//] I use this type of pedagogy as a political act […].*

*(P1)*

However, participants still have to face stress and anxiety when they foresee possible situations, contexts, or people that may wield some kind of discrimination against them, not only homophobia.

*So, for me it is sexual orientation in the workplace, it has caused me very serious problems, and that conditions me nowadays [//] but it has conditioned me throughout my whole life, because I still stuffer aftereffects of the great anxiety episodes I have experienced since I was a child […].*

*(P5)*

Finally, throughout the data provided by the participants, two factors stand out as being closely related to wellbeing. The first corresponds to the stability of their contract. The higher the position, the greater their level of wellbeing, especially with regard to the microsystem. In this way, full-professor participants perceived things related to self-concept and personal and professional development more positively, contrary to what happens with teachers who are non-permanent. The second factor is represented by their command of the foreign language, so that teachers with a greater linguistic level or those teaching in their own mother tongue have a better perception of their wellbeing.

*Not having a permanent position makes me feel highly stressed [!], it has negative effects mainly on my mental health.*

*(P3)*

*I think that teaching in a language you master can make you feel more comfortable with yourself, because you do not need to pay so much attention to the words you say, so you can speak freely as you know you’re not going to make a mistake, and you won’t be held in contempt by your students.*

*(P2)*

## 4. Discussion

In this section, we discuss the most relevant results of our research as they relate to those previously stated by the scientific literature on queer university language teachers’ wellbeing. In this way, we accomplish the objective of our research, which was to examine the interplay between queerness and the wellbeing of foreign language teacher trainers in a Spanish context from an ecological perspective.

To begin with, data related to the microsystem confirm that self-acceptance stands as one of the core factors that determines our participants’ teacher wellbeing. This is permanently influenced over time by the relationships they establish with other variables belonging to the rest of the ecological sub-systems (meso-, exo-, macro-, and chronosystems), considerably determining both the objective and subjective wellbeing of the participants. Among the variables mentioned above, the following leap out: relationships with the family; work relationships (management teams, colleagues and students); self-acceptance of sexual orientation; the very idiosyncrasy of the university ecology that makes it an exceedingly competitive environment; and high-demanding working conditions that negatively affect the balance between personal and work life; as well as the role that foreign language plays in the participants’ self-acceptance both in the private and professional spheres.

As we have seen throughout this study, most of the participants display a high level of wellbeing in terms of self-acceptance. However, certain episodes of subtle and blatant discrimination suffered by the participants in the course of their lives, from childhood to the current stage, have had a negative impact that reaches the present moment. These results are consistent with those revealed in the studies by Badgett et al. [44] and DeSouza et al. [43], confirming that stigmatizing events in the lives of queer male teachers are the basis for a high level of emotional stress. Thus, the participants have developed a state of constant alarm that produces stress and anxiety in the face of what they perceive to be a threat. This phenomenon often has negative consequences on the stability of their level of self-acceptance and concerns both their work and personal environment. In this sense, studies such as those of Spencer and Patrick [56], Domínguez-Fuentes et al. [40], Schmitt et al. [55], and Molero et al. [9] confirm the high negative impact of discrimination on self-acceptance and psychological wellbeing. These authors also show that self-acceptance improves over time after facing conflicts and discrimination thanks to the development of resilience and the establishment of positive relationships, which is also related to a greater mastery of the environment, as we have also corroborated with the results of our study. It is worth highlighting the role that acceptance of sexual orientation plays in the participants’ personal and professional growth. They manifest higher signs of wellbeing when they openly express their sexual orientation in the workplace, which parallels the studies of Schmidt and Kurdek [57] and Morris et al. [58].

With regard to the mesosystem, the great importance that participants attribute to maintaining positive relationships in their work and personal environment stands out. In this sense, our data corroborate previous studies [3,22,30] regarding the impact of the relationships that teachers establish in the work environment on their perception of wellbeing. That being said, the vast majority of our participants declared having suffered some kind of discrimination. The nature of this discrimination in the university setting has been jokes, discriminatory treatment by colleagues, abuse, obstacles to promotion in the career, or obstacles to job promotion, as also stated in the study carried out by FELGT-COGAM [39] in work placements throughout Spain.

The homotransphobic discrimination suffered by the participants influences both their interpersonal relationships and their sense of freedom to give opinion in groups, not allowing them to fully develop for the fear of being blamed or not understood. They feel isolation and frustration with their social relationships, thus avoiding deep and stable involvement with other people [84]. Most of the time, they only feel safe around close acquaintances with whom the participants can freely express their sexual orientation, thus having a positive effect on relationships and psychological balance. This result correlates with the study of Bostwick et al. [8] that associates sexual orientation discrimination with higher rates of mental health disorders suffered by gay, lesbian, and bisexual groups as compared with heterosexuals. This also aligns with Moya and Moya’s [10] research that demonstrates that LGTBQ+ people in Spain still perceive and experience greater discrimination in the workplace, which leads to work stress, mental disorders, and depression. More specifically, social prejudices, such as the sense of exclusion and non-belonging, worsened during their adolescence due to harassment episodes, causing a significant erosion of their wellbeing and their ways of interacting. This also accords with Meyer’s study [17], which focused on teacher expression and sexual orientation in schools of Canada. Even though the passing of time has lowered this negative impact on their lives, as already proven by Molero et al. [9] and Domínguez-Fuentes et al. [40], our study findings are consistent with those of Moya and Moya [10], as they call attention to the interplay between sexual orientation and discrimination in the workplace, which is a far more complex phenomenon, and might be either neglected or trivialized.

Despite the negativity they have experienced, not all the participants perceive themselves as victims. They visualize themselves as empowered agents—i.e., LGTBIQ+ activists who demand justice and equal treatment and opportunities within the discriminatory context. This broadly supports the work of other studies in this area [75,76,77,78]. Therefore, openly expressing homosexuality in class represents for them a pedagogical bet in which they assume the role of models for foreign language pre-service teachers. Thus, they denounce that silence and invisibility are subtle homophobic violence, claiming the importance of being honest in the classroom and in life. This political positioning implies benefits for their self-esteem, self-concept, and teacher wellbeing, as well as in the classroom dynamics and learning processes. All of this results in a higher level of job satisfaction and a healthier and closer connection with the students, paralleling what has been stated by Lander [5], Gray [16], and Nelson [68]. Our participants’ ability to manage the environment identifies them as people with the capacity for autonomy and self-determination, despite the stress and anxiety that multiple social and work pressures may imply, which, according to Ryff [84,85], corresponds to a high degree of psychological wellbeing.

As we have been able to corroborate, most of the participants show a great capacity for resilience and post-traumatic growth [94], a confirmed key factor for teacher wellbeing as argued by Tennen and Affleck [94]. Consequently, many of them usually resort to coping mechanisms to deal with daily stressors, such as the use of humor [95] and the relativization and vindication of sexual orientation in the form of activism, as defended by Carver et al. [95]. These refer, respectively, to distancing and seeking social support according to Folkman et al. [96]. This demonstrates an adequate capacity to function in diverse and adverse contexts, as well as an ability to create and select favorable environments in which to grow personally and professionally. This could be construed as an example of post-traumatic growth making sense of negative events as an adaptive tool [94], restoring life’s meaning and helping to redefine oneself. However, queer male university teachers need greater social support, as well as recognition by society and institutions so that their resilience capacity is not taken for granted or trivialized, as confirmed by Jin et al. [4]. In this sense, Margolis et al. [97] denounce the idea that sometimes the teaching institution is the only one that benefits as a result of an abuse of the resilience capacity of its workers.

Next, we discuss the data regarding the macrosystem. With regard to the specificity of their area of expertise, all the participants perceive the influence they exert on their students (Primary and Secondary teacher trainees) as an element that promotes their wellbeing. They especially focus on their role as queer teacher models, a fact that Lander [5], Clarke [66], and Donahue [98] also confirmed in their contexts. Another exponentially significant factor that permeates teacher wellbeing is the positive conception of language as an instrument of social interaction. That being said, foreign languages foster teachers’ personal and professional flourishing. Subsequently, teaching acts as a liaison between cultures and diverse people, assuming different ways of understanding the world, while contributing to the reflection on one’s own culture and language.

All things considered, university language teachers who suffer or have suffered discrimination due to their sexual orientation require greater social support. This is based both on the stress that arises from fear or from a constant state of alarm in the face of possible discrimination on account of their sexual orientation, as well as the insecurity that springs up from the physical and emotional challenges entailed by foreign language teaching.

Wieczorek’s [99] findings revealed the presence of inhibitions and feelings of low self-esteem related to the level of linguistic competence of the investigated language teachers. In this sense, the fear of making a linguistic error when speaking or writing generates certain linguistic anxiety that is closely linked to other facets of their identity, such as sexual orientation. In this vein, some participants identified the perceived fear of rejection on the part of their students for being queer. These fluctuations between identity and language skills, coupled with the ongoing stress generated by the relationships with students, colleagues, and management teams, as well as poor working conditions, place their wellbeing at risk. These facts countersign the conclusions derived from other closely related studies [2,99,100].

As for the chronosystem, results confirm that the homotransphobic episodes suffered in the different stages of the private and professional lives of our participants have not only acted as milestones in their wellbeing but have also enhanced their capacity of resilience, in agreement with the conclusions drawn by Roffey [31], Molero et al. [9], Spencer and Patrick [56], and Domínguez-Fuentes et al. [40]. Likewise, the imbalance between private and work life is maintained over time as one of the factors that negatively influences teacher wellbeing. At the same time, the participants in our study handle their sexual orientation in different ways in their work. The reason lies fundamentally in the dependency relationship that is established with the cultural and temporal context, as underpinned by Griffin’s pioneering research [80] and later studies such as those of Aelterman et al. [22], Price and McCallum [19], and van Lier [100]. They all describe wellbeing as a psycho-social construct that is dynamic, of emerging character, with a constant state of being, and strongly marked by the context. Additionally, our study shows that the stability of the contract and the command of the language being taught are wellbeing indicators [79,80,81]. The linguistic command of the foreign language is also an indicator of wellbeing: the greater the linguistic domain, the greater the wellbeing that is evidenced in the studies of Lander and in Lin et al., respectively [5,6].

To conclude, we must highlight the limitations of this study in terms of the size and sample. However, we consider that the results can generate a preliminary theoretical basis when it comes to this understudied topic. With this, we open the way to new theories and contrasting study models that can be useful for evaluating and determining the queer university language teacher’s wellbeing. These may be aimed at, on the one hand, improving their working conditions, and, on the other hand, creating ongoing teacher training programs with a special focus on self-acceptance and personal growth, flourishing, positive relationships, and stress management techniques, as well as on recycling and fostering foreign language linguistic and social skills.

## 5. Conclusions

Conducting a qualitative study based on an ecological model has allowed us to understand the interrelationship of the different subsystems, as well as the responses that enable these teachers to assess the barriers and achievements in their private and professional lives. It is of special interest to have verified that sexual orientation is a relevant aspect of identity in all subsystems, thus becoming not only an element of personal identity but also an element of politics, always mediated by culture. At the same time, it has had a negative impact on the teacher wellbeing of the cohort, and their psychological capital has proven to be pivotal for overcoming the problems derived from discrimination in the private and professional spheres.

Likewise, the foreign language, as an area of professional knowledge, has played a leading positive role in the participants’ psychosocial capital for the management and self-acceptance of one’s sexual identity and, ultimately, a role in being able to flourish personally and professionally.

We can conclude this paper by saying that it has been demonstrated that teacher wellbeing is a dynamic psychosocial construct that unfolds over time, strongly linked to the (inter)personal, institutional, cultural, and political context of teachers. These results serve as fodder for further research on this topic. We still need more evidence to have enough field knowledge of what contributes to the teacher wellbeing of LGTBIQ+ language teachers in different geographical contexts and levels. This learning should also be transferred to decision makers in education and politics and to management teams in an effort to make them allies in the support and initial and continuous training of teachers and the promotion of their wellbeing.

## Figures and Tables

**Table 1:** Informants.

Subject	Sexual Orientation	Academic Training	Teaching Experience	Political Trend	Religion	Marital Status	Children
P1: Junior English lecturer aged 29, English C1, Portuguese B1	Gay	PhD, MA, MA, BA	2 years	Left	Agnostic	Common-law couple	0
P2: Junior French lecturer aged 32, French C2, English C1	Gay	PhD, MA, MA, BA	4 years	Left	Non-practicing religious	Common-law couple	0
P3: Junior English lecturer aged 39, English C2, Spanish C1	Gay	PhD, MA, BA	2 years	None	Atheistic	Common-law couple	0
P4: Senior English lecturer aged 43, English C2, German B1, French B1	Gay	PhD, MA, MA, BA, BA, BA	18 years	Center	Agnostic	Single	0
P5: Senior English lecturer aged 43, English C2, French B2	Queer	PhD, MA, MA, BA, BA, BA	18 years	Center	Agnostic	Single	0
P6: Senior English lecturer aged 52, English C2	Bisexual	PhD, MA, BA	25 years	Left	None	Single	0
P7: Senior Spanish lecturer aged 56, French C1, Italian B2	Gay	PhD, MA, BA, BA	32 years	Left	None	Married	1

(Own elaboration).

**Table 2:** Thematic areas.

Thematic Areas
1. Aspects related to individual psychology, such as self-acceptance or autonomy.
2. Aspects related to private and professional relations with other people.
3. Private and professional aspects related to the management of the environment.
4. Private and professional aspects related to life objectives and personal development.

(Own elaboration).

**Table 3:** Links and interplay of sexual orientation in teacher wellbeing.

Sub-Systems	Themes	Values	Participants
Microsystem	Self-concept as LGTBIQ+ person	Positive after evolution in time	All
	Sexual orientation in the identity spectrum	Intersectionality	All
		Against hyper-queer identification	P4, P5
		Against identity labelling	P5, P6
	Self-commitment	Strong value	P1
	LGTBIQ+ models for identification	In education field	P1
		Sentimental partner	P1
		People in show business	P1, P5
		Absence, irrelevance	P2, P3, P4, P6, P7
	Personal development	Positive stimulus	P1, P2, P5, P6, P7
		Absence	P3, P4
	Life objectives	Building of a family	P7
		Irrelevant or blurred in time	P1, P2, P3, P4, P5, P6
	Influence on students in their roles as LGTBIQ+ teachers	Role models as queer people: honesty, freedom	All, but P3
		Inclusion of queer perspective in teaching	P1, P2, P4, P5, P7
	Dealing with LGTBIQ+ issues in the classroom	Students: general support with sporadic problems	All, but P3
Mesosystem	Influence that other people exert on participants’ lives	Relevant	All
	Expressing opinion in public	Problematic	P1
	Impact of sexual orientation in private relations	Sense of non-belonging	P1
		Under suspicion	P5
		Problems with acquaintances	P7
	Impact of sexual orientation on school life	Homotransphobic bullying	All
		From problem to opportunity for social justice	All, but P3
	Impact of sexual orientation on family life	Problems, non-acceptance	All
		Problems in rural world	P1, P4, P5
		Need for education: against structures, not against members of the family	P1, P5
	Perspectives on LGTBIQ+ people	Recognition and support	All
	Interplay of sexual orientation and university life	Management teams: harassment	P5, P6
		Colleagues: harassment, problems	P1, P2, P4, P5, P6
		Research: rejection of queer topics	P4, P5
		Knowledge transfer: positive university associations	P1
Macrosystem	LGTBIQ+ legislation in Spain	Positive, but insufficient	All, but P3
		Strong need for education	All, but P3
	Interplay of sexual orientation and the area of foreign languages	Openness to cultural and identity otherness	All, but P3
Chronosystem	Transversal to all the sub-systems	Resilience, transition from negative to positive	All

(Own elaboration).

## Data Availability

Data supporting reported results can be obtained by mailing the authors.
